# Toward a Molecular Understanding of the Interaction of Dual Specificity Phosphatases with Substrates: Insights from Structure-Based Modeling and High Throughput Screening

**DOI:** 10.2174/092986708785909003

**Published:** 2008-10

**Authors:** Ahmet Bakan, John S Lazo, Peter Wipf, Kay M Brummond, Ivet Bahar

**Affiliations:** 1 Department of Computational Biology, University of Pittsburgh, Pittsburgh, Pennsylvania, USA; 2 Department of Pharmacology and Chemical Biology, University of Pittsburgh, Pittsburgh, Pennsylvania, USA; 3 Department of Chemistry, University of Pittsburgh, Pittsburgh, Pennsylvania, USA; 4 University of Pittsburgh Drug Discovery Institute, University of Pittsburgh, Pittsburgh, Pennsylvania, USA

**Keywords:** Dual-specificity phosphatases, computer-aided drug discovery, high throughput screening, structure-based virtual screening, molecular docking, intrinsic dynamics, focused library design.

## Abstract

Dual-specificity phosphatases (DSPs) are important, but poorly understood, cell signaling enzymes that remove phosphate groups from tyrosine and serine/threonine residues on their substrate. Deregulation of DSPs has been implicated in cancer, obesity, diabetes, inflammation, and Alzheimer’s disease. Due to their biological and biomedical significance, DSPs have increasingly become the subject of drug discovery high-throughput screening (HTS) and focused compound library development efforts. Progress in identifying selective and potent DSP inhibitors has, however, been restricted by the lack of sufficient structural data on inhibitor-bound DSPs. The shallow, almost flat, substrate binding sites in DSPs have been a major factor in hampering the rational design and the experimental development of active site inhibitors. Recent experimental and virtual HTS studies, as well as advances in molecular modeling, provide new insights into the potential mechanisms for substrate recognition and binding by this important class of enzymes. We present herein an overview of the progress, along with a brief description of applications to two types of DSPs: Cdc25 and MAP kinase phosphatase (MKP) family members. In particular, we focus on combined computational and experimental efforts for designing Cdc25B and MKP-1 inhibitors and understanding their mechanisms of interactions with their target proteins. These studies emphasize the utility of developing computational models and methods that meet the two major challenges currently faced in structure-based *in silico* design of lead compounds: the conformational flexibility of the target protein and the entropic contribution to the selection and stabilization of particular bound conformers.

## INTRODUCTION

Dual-specificity phosphatases (DSPs) are important signal transduction enzymes that regulate various cellular processes including cell division, growth differentiation, proliferation and apoptosis, in coordination with protein kinases [1]. They form a subclass of the protein tyrosine phosphatase (PTP) superfamily, which are distinguished by the active-site signature motif HC*X*_5_R at the phosphatase (PTPase) loop [2]. Forty of the 107 PTP genes in the human genome encode DSPs [3]. Deregulation of DSP protein expression levels has been implicated in at least ten cancer types, obesity, diabetes, inflammation, and Alzheimer’s disease [1, 4]. This therapeutic spectrum has led to a growing medicinal chemistry interest in exploring DSPs.

DSPs are functionally defined by their ability to catalyze the removal of two covalently attached phosphate groups from tyrosine and serine/threonine residues on the same substrate. This activity is achieved *via *the PTPase catalytic mechanism at a relatively shallow active site; presumably, this shape is required for accommodating the phosphorylated serine/threonine residues which, in contrast to phosphorylated tyrosine, only extend slightly beyond the peptide backbone [5]. DSP inhibitors have been identified in natural product collections [6, 7], diversity-oriented chemical libraries [8-10] as well as in larger scale drug-like compound libraries [11]. These compounds have not been widely used as molecular probes or lead compounds in part because of their lack of potency, redox properties, and inadequate phosphatase selectivity, despite some limited synthetic analog follow up studies [12]. Fig. (**1**) presents an overview of the ensemble of proteins targeted by approved drugs, retrieved from DrugBank [13], which showcases the small fraction of protein phosphatases and the lack of representation of DSPs.

Computational methods have become ubiquitous in all aspects of drug discovery [15], including structure-based modeling studies broadly used in lead discovery and optimization [16]. Applications of structure-based modeling to DSPs have been limited, however, due to the lack of structural data. Over the past two years, the number of known distinct DSP structures has more than doubled, providing the framework for developing structure-based modeling for compounds of therapeutic interest. Fig. (**2**) illustrates the catalytic domain of a DSP, cell division cycle 25B (Cdc25B) phosphatase, in comparison to two extensively studied cell signaling enzymes, protein tyrosine phosphatase 1B (PTP1B) [17] and cyclin-dependent kinase 2 (Cdk2) [18]. PTP1B and Cdk2 structures have a deep groove at their active sites, which ensures the tight binding of small molecule ligands, and allows for the design of selective inhibitors. The lack of analogous active site features in DSPs increases the challenge for the design of small molecule inhibitors.

It is reasonable to hypothesize that improving our understanding of the interactions of DSPs with their substrates and inhibitors could accelerate the discovery and development of therapeutic agents. Here we will focus on two therapeutically important groups of the DSPs: Cdc25s and MAP kinase phosphatases (MKPs), and present an overview of recent advances in understanding their interactions with ligands at the molecular level. We summarize the results from high-throughput methods for lead discovery and chemical synthesis and virtual design methods for lead optimization applied to these DSPs. Two major challenges in the *in silico* design of lead compounds that target these DSPs, which are common with most molecular docking efforts, are modeling the conformational flexibility of the protein and accounting for the entropic effects that stabilize bound inhibitor conformations. Finally, we discuss prospects toward addressing these challenges by applying advances in protein structural dynamics modeling.

## CDC25 PHOSPHATASES: STRUCTURE, FUNCTION AND INTERACTIONS

### Overview of Function, Sequence and Structure of Cdc25 Phosphatases

Cdc25 phosphatases are key regulators of the cell division cycle and modify Cdks [19]. The human genome encodes three Cdc25 isoforms, designated by the suffixes A, B, and C. In the normal cell division, they catalyze the activation of Cdk/Cyclin complexes leading to cell cycle progression, e.g., Cdc25B activates Cdk2-pTpY/CycA contributing to early G2 phase progression. In addition, the inactivation of Cdc25s by checkpoint kinases (Chk1 and Chk2) in response to damage to or improper replication of DNA results in cell cycle arrest [20]. In the context of cell division progression, the A and B isoforms have been reported as potential oncogenes [21], being overexpressed in more than ten types of human cancer, including prostate [22] and breast [23] cancers.

**Fig. (2):**
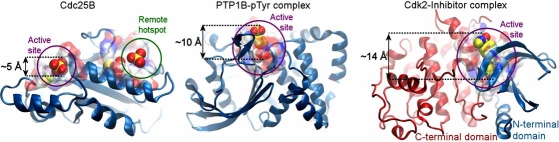
**Comparison of the active sites of Cdc25B, PTP1B and Cdk2.** The active site on Cdc25B is rather shallow, compared to the deep pockets that permit the insertion of ligands in PTP1B and Cdk2. A remote hot-spot is shown in the Cdc25B structure. Recent identification of a hotspot interaction between Cdc25B and its native substrate, the Cdk2/CycA complex, has shifted some drug discovery efforts targeting Cdc25B from the active site to the remote hotspot region.

**Fig. (3):**
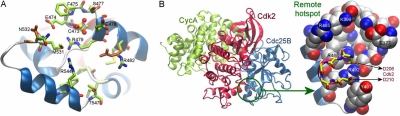
**Active site and remote hotspots at the Cdc25B catalytic domain. A.** Cdc25B active site. A sulfate is bound to the catalytic site cavity. Different side-chain orientations might affect the outcome of inhibitor docking studies (PDB IDs: 1QB0 colored green, 1YMK colored orange). **B.** Computer model for the Cdc25B-Cdk2/CycA ternary complex and remote hotspot interactions at the interface between Cdc25B and Cdk2.

The Cdc25 encoding sequences are 460 to 550 amino acids long and are described in terms of N-terminal and C-terminal functional regions. The N-terminal region contains the regulatory sites; the C-terminal region, around 200 residues long, encodes the catalytic domain. The regulatory domain shows high sequence variability among the isoforms including alternative splice variants, whereas 85% of the amino acids in the catalytic domain are identical. The catalytic domain of Cdc25 is topologically unique from that of other PTPs (Fig. **2**) and assumes almost identical structures in the isoforms A [24] and B [25] (0.8Å root-mean-square deviation (RMSD) in their 148 C^α^-coordinates) with the exception of the disordered C-terminal α-helix in isoform A. Several high-resolution structures of the catalytic domain of Cdc25B have been determined, including single residue mutations [26] or different oxidation states of the catalytic cysteine [27]. These structures show minor conformational differences in the side chains of solvent-exposed residues. This conformational variability, illustrated for Arg482 and Asn532 in Fig. **3A**, affects the binding pose of the ligand at the active site of Cdc25B.

### Cdc25B Substrate Interactions: Enzyme Inhibitors and High Throughput Screening (HTS)

A general challenge in developing effective small molecule inhibitors is the identification of an appropriate starting or lead structure. Such compounds are often identified serendipitously or by systematic experimental or computational HTS of small-molecule libraries. The first Cdc25 inhibitors, the dnacins and the dysidiolides, have been reported more than a decade ago [1, 28]. Since then, a variety of different chemical classes of Cdc25 inhibitors have been identified by traditional or HTS methods. These include lipophilic acids, oxazoles, sterols, polyphenols, terpenoids, indoles, and quinones [28, 29]. HTS strategies to identify small molecule inhibitors of Cdc25s have generally followed the approaches used for other PTPs [30]. Either low or high throughput screens have been developed using recombinant protein with a variety of small molecule substrates, including *p*-nitrophenyl phosphate, 6,8-difluoro-4-methylumbelliferyl phosphate, fluorescein diphosphate or O-methyl-fluorescein phosphate. These artificial substrates are selected in preference to the native substrate to avoid the need to generate both a Cdk and a Cyclin and then phosphorylating the complex with a kinase. Recent HTS protocols and screening results against Cdc25B and MKPs that were sponsored by the NIH Roadmap Molecular Libraries Initiative, including data from the University of Pittsburgh Molecular Libraries Screening Center, can be found online (PubChem; http:// pubchem.ncbi.nlm.nih.gov/).

### Cdc25B Substrate Interactions: Protein-Protein Interfaces

The shallow nature of the Cdc25 active site suggests that a large interfacial area is involved in recognizing Cdk. In view of the difficulty of specifically targeting this site, attention has turned in recent years to alternative regions in the enzyme. One strategy focuses on identifying single amino acids that are critical for the phosphatase-substrate interaction, so-called hotspots, at protein-protein interfaces [31, 32]. In the case of Cdc25B, 13 residues were selected, based on their proximity to the active site, conservation in Cdc25 isoforms, and potential geometric and physicochemical complementarity to the Cdk2 structure. Site-directed mutagenesis was used to determine the importance of each of the 13 residues for Cdc25B-Cdk2/CycA interaction. [33]. Mutations of Arg488 and Tyr497 reduced both the *in vitro* and *in vivo* dephosphorylation of Cdk2-pTpY/CycA by Cdc25B. Computational modeling of the Cdc25B-Cdk2/CycA tertiary complex by Rudolph and coworkers further improved our understanding of the mechanism of substrate recognition by Cdc25B (Fig. **3B**) [34]. Binding experiments of mutants selected after computer modeling identified additional key residues (Arg492 on Cdc25B and Asp206 and Asp210 on Cdk2) that mediate the protein-protein association. The Arg492-Asp206 interaction was found to make the largest contribution (3.8 kcal/mol) to the total free energy of binding, followed by Arg488-Asp206 (2.8 kcal/mol). Structural characterization of this region revealed several features that support the feasibility of developing Cdc25B-Cdk2/CycA interaction inhibitors. A pocket located next to the hotspot arginines remained unoccupied by Cdk2, and was suggested by computational models to serve as an anchor for small molecule binding. The hotspot residues and this nearby pocket provided approximately 350 Å^2^ solvent accessible surface area (220 Å^2^ non-polar and 130 Å^2^ polar) [35] and featured several potential hydrogen bond acceptors and donors (backbone atoms of Leu378, Asp397, Arg485, Pro503 and Met505, and side-chain atoms of Glu377, Lys399, Arg485, Arg488, Glu489 and Arg492) (Fig. **3B**). The volume of the pocket was approximately 200 Å^3^, which was large enough to accommodate a fragment-like molecule (MW < 250). Amino acid variations at two positions in the pocket, Phe386 (Cdc25A:Tyr344 and Cdc25C:Cys290) and Met505 (Cdc25A:Leu362 and Cdc25C:Leu409) further supported the feasibility of anchoring selective inhibitors at this site.

## MKPS: STRUCTURE, FUNCTION AND INTERACTIONS

### Overview of Function, Sequence, and Structure Characteristics of MKPs

MKPs dephosphorylate and inactivate mitogen-activated protein (MAP) kinases [36]. Their substrates include p38 kinases (p38s), c-Jun amino-terminal kinases (JNKs) and extracellular signaling-related kinases (ERKs) [37]. Members of the MKP family show tissue specific expression patterns and tight growth/mitogen/stress regulated transcriptional induction profiles [38]. At least five of the 11 family members have been implicated with various cancer types [39]. Among these, MKP-1, the prototypical member, and the structurally well-characterized MKP-3 have been most closely examined. MKP-1 is a nuclear protein and shows selectivity for p38s and JNKs over ERKs. Its expression levels are elevated in at least eight cancer types and correlate with the progression of breast [40] and lung [41] cancers. MKP-3 is a cytosolic phosphatase with preference for ERKs over other MAP kinases. Overexpression of MKP-3 is associated with lung [42] and pancreatic [43] cancers.

**Fig. (4):**
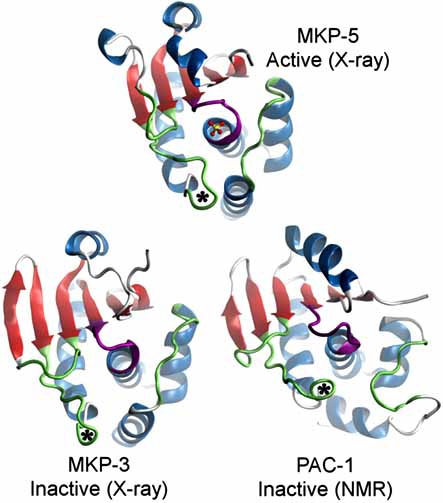
**MKP CD domain structures of active and inactive states.** The active site loops display substantial changes between active and inactive states. General acid loops are marked with an asterisk. Coloring is according to the sequence alignment shown in Fig. (**5**).

MKPs vary in length from 180 to 660 amino acids and all possess a canonical tyrosine phosphatase domain [44]. Most of the members also possess an N-terminal substrate binding domain (BD) that facilitates substrate recognition and specificity. A list of MKP catalytic domain structures resolved to date is given in Table **1**, along with Cdc25A and Cdc25B catalytic domains. These DSPs exhibit at least 40% sequence identity with respect to the catalytic domains of MKP-1 and MKP-3 (Table **1**). Selected structures are shown in Fig. (**4**), and their sequence alignments are presented in Fig. (**5**). MKP catalytic domains share the same fold with PTPs, - a five stranded β-sheet (six stranded in the inactive state of PTPase loop) surrounded by four α-helices on one side and one α-helix on the other (Fig. **4**). The MKP structures resolved in the inactive state of the PTPase loop, MKP-3 [45] and PAC-1 [46], are known to adopt the active PTPase loop conformation upon binding their substrates. On the other hand, MKP-5 [47] and VH3 [48] adopt the active state PTPase loop conformation even in the absence of bound substrate, consistent with the intrinsic ability of some enzymes to sample conformations that facilitate ligand binding even in the unbound state [49]. In addition, BD structures of MKP-3 [50] and MKP-5 [51] have been resolved.

### Substrate Recognition and Catalytic Activation: Alternate Mechanisms for Inhibition

Substrate recognition is achieved by the BD of MKPs. In the case of MKP-1 [52], MKP-3 [53], and PAC-1 [54], allosterically bound substrate triggers a catalytic activation and induces a movement of about 5 Å in the acidic loop (Fig. **4**). This geometric adjustment provides two potential inhibition mechanisms as alternatives to the conventional direct catalytic PTP inhibition. The first would involve the inhibition of substrate recognition, similar to the mechanism proposed above for Cdc25B-Cdk2/CycA. The second would target the obstruction of the allosteric mechanism of activation. Such an inhibitory action has been achieved for PTP1B *via* the restriction of a catalytically important loop from adopting an active state conformation upon binding a small molecule [55]. We note that MKP-3 preferentially dephosphorylates ERK2, and in addition to the resolved structures of the catalytic domain and BD, a structure of ERK2 complexed with the kinase interaction motif from MKP-3 BD has been determined [56]. The specific regions of MKP-3 that interact with ERK2 have been elucidated in systematic mutation and deletion analyses [57]; and finally the results from H/D exchange experiments [58] have been used to construct a structural model of the MKP-3/ERK2 complex. All of these data suggest that a further examination of the binding surface and dynamics of MKP-3 is warranted and could assist in identifying novel sites for enzyme inhibition.

## COMPUTATIONAL MODELING OF PROTEIN-INHIBITOR INTERACTIONS

### Modeling Protein-Ligand Interactions: Importance of Entropic Effects in Defining Binding Affinities

Molecular docking enables the rapid investigation of molecular recognition at an atomic scale [59]. With the steady increase in the number of experimentally determined protein structures, docking, including docking virtual libraries, is becoming widely used in drug discovery for predicting inhibitor/substrate binding modes, especially in the absence of a co-crystal structure. A comprehensive overview of current docking methods can be found in a recent review [60]. The critical assessment of a series of docking programs and a large set of scoring functions used in computer-aided drug discovery led to two conclusions: docking software are useful for distinguishing active compounds from a large set of relevant decoys, and (ii) scoring functions are less successful in selecting the crystallographic conformation of a ligand from a set of docking poses, and cannot provide an accurate ranking of the relative binding affinities of different ligands which limits their utility in lead optimization [61]. 

The deficiency of scoring functions is essentially attributed to the insufficient consideration of entropic effects [62]. Due to computational efficiency considerations, molecular docking simulations are usually conducted by employing an optimal conformation of the target protein; for example, a known structure of the protein complexed with other ligands. The small molecule is allowed to undergo rigid body and internal structural changes to optimize its interaction with the fixed protein or a fixed backbone conformation with a few rotatable side chains. This level of approximation based on a single, ‘optimal’ bound conformation, results in systematic errors in binding free energy calculations [63]. Current docking algorithms predict an incorrect binding pose for 50-70% of all ligands when only a single fixed receptor conformation is considered [64], and models with increased levels of complexity to capture conformational variations have been suggested [65]. In 2007, two notable studies that take rigorous account of entropy showed significant success in predicting binding affinities: Gilson and coworkers analyzed the association of amprenavir with HIV-1 protease using the Mining Minima method, which calculates binding free energy as the difference between the standard chemical potentials in the bound and unbound states using multiple conformations of the ligand at each state [66]. Dill and coworkers, on the other hand, studied T4 lysozyme ligands using an alchemical free energy calculation method in explicit solvent [67]. They showed that the consideration of the multiple binding modes of ligand and multiple side-chain orientations in calculations gradually increases the accuracy of the predictions. 

### The Shape, not only the Depth, of the Free Energy Surface, Defines Binding Affinities

Recently, Ruvinksy showed that the energy well associated with the experimentally observed binding mode of ligands is broader than all energy wells associated with non-native binding poses [68]. In other words, the bound conformation accommodates slight variations in the binding pose without affecting (weakening) protein-ligand interaction energy (enthalpy), and this variability, or associated favorable entropy, helps in lowering the free energy of binding. This finding is consistent with the above-mentioned studies of the Gilson and Dill groups, which point to the importance of entropic effects in stabilizing bound conformers. A similar *ensemble modeling* approach that takes into account the width of the energy wells was recently adopted to explain the differences in the catalytic rates of cytochrome P450 orthologs that process the same substrates [69].

These results provide us with insights into how one should select the most likely docking poses. Using molecular docking programs, an improved accuracy of predictions can be achieved by clustering analysis of a *population* of docking poses. Thus, it is possible to define various bound ‘states’ (clusters), comprised each of the multiple ‘microstates’ (conformations). The evaluation of the binding energy at each state can then be based both on the energetic interaction *and* on the population of microstates in each state. Indeed the interaction energies, usually accounted for by the scoring functions of the docking software, simply represent the enthalpic contribution to the binding free energy, and the entropic contributions scale with the size of the populations of microstates. A practical shortcut that may improve docking calculations was shown to utilize multiple fixed receptor conformations, i.e. perform the simulations with an ensemble of structures, either experimentally determined by crystallography or NMR, or computationally generated [64]. Such approaches are expected to be particularly useful in the study and design of DSP inhibitors, because the shallow active sites of these enzymes provide considerable conformational freedom to bound small-molecule ligands.

### Modeling Functional Dynamics of Proteins: Flexible Docking

To make an accurate assessment of the ensemble of microstates accessible in a given bound state, it is important to consider the conformational flexibility of the target protein. A major challenge in virtual docking studies, however, is to accurately consider target protein flexibility without significantly increasing the computational (time) cost [70]. We note that the Abagyan laboratory has made significant progress in developing docking methods and software that incorporate protein flexibility [64, 71,72].

Recent years have seen a surge in the number of studies exploring the conformational dynamics of proteins using coarse-grained normal mode analysis (NMA) [73] with the realization that the NMA of a given protein structure (e.g., apo form, or ligand-bound) can provide information on alternative functional conformations most likely to be assumed by the same protein in the presence of other ligands, or under other conditions. The beauty of such analyses is the robustness of low frequency modes, which can be captured even by low resolution models such as ENMs. The underlying hypothesis in these studies, now confirmed in many experiments [74, 75], is that each protein has an ‘intrinsic dynamics’ favored by its 3-dimensional structure, i.e. it samples a well-defined ensemble of conformations in the neighborhood of the native state that are structurally defined; the experimentally observed alternative forms are simply those conformations that are intrinsically accessible and stabilized by substrate or ligand binding [49]. This permits one to generate an ensemble of conformers for each target protein, which can be utilized in docking simulations with selected lead compounds. Alternatively, ENM-based methods permit one to make an assessment of conformational changes that are easily accessible and those requiring excessive strains, when evaluating the alternative bound conformations of target proteins. In fact, normal mode analysis is now thought to be a prerequisite for drug design [76].

## COMPUTATIONAL MODELING OF DSP-INHIBITOR INTERACTIONS

### Modeling Cdc25B Interactions with Inhibitors: Multiple Modes of Binding

Several groups have performed molecular docking studies to help clarify the nature of catalytic inhibitor interactions with DSPs. We examined two chemotypes, 2,3-bis-[2-hydroxyethylsulfanyl]-[1,4]naphthoquinone (NSC 95397) and 3-benzoyl-naphtho[1,2-*b*]furan-4,5-dione (5169131), as potential lead compounds for Cdc25B inhibition [9, 11]. The Cdc25B active site was targeted in these docking simulations, revealing a potential small molecule interaction with Arg482 and Arg544 side chains (Fig. **3A**). In another study, several inhibitors of Cdc25B were examined using two docking programs and the results were supported by structure-activity relationship and site-directed mutagenesis data and guided the design of more potent inhibitors [77]. More recently, Park *et al.* studied ligand binding to a relaxed conformation of the Cdc25B catalytic domain generated by molecular dynamics simulations and proposed a new binding pose for NSC 95397 [78]. The different binding poses are likely from the result of the combined effects of the selected docking algorithms, scoring criteria, and receptor conformations, further complicated by the shallow shape of the Cdc25B active site. It is also possible that the enzyme accommodates more than one binding mode, but this has not yet been verified by any structural data. A systematic analysis of the ensemble of the most favorable binding modes using clustering methods would appear to be a plausible computational approach toward gaining further insights into the possible modes of inhibitor binding to Cdc25B.

A more recent application of molecular docking was to identify new catalytic inhibitors of Cdc25B in a multistep virtual screening procedure [79]. The research group selected 1,500 compounds from a set of 313,000 based on their complementarities to the Cdc25B active site. These compounds were subjected to an *in vitro* enzymatic assay to yield 99 compounds with 20% inhibitory activity at 100 μM concentration. Two compounds with the highest *in vitro* potency (IC_50_ values of 13 ± 0.5 μM and 19 ± 1.3 μM) were also shown to inhibit the proliferation of HeLa cells in a concentration dependent manner (IC_50_ = 15.8 ± 1.8 μM and 3.6 ± 1.2 μM). 

### Assessment of MKP-1 and Inhibitor Interactions Using Ensemble Modeling

MKP-inhibitor interactions were recently modeled for the first time upon the determination of the active structure of MKP-5 [47]. Members of a pyrrole carboxamide (focused) library were investigated to gain insights into possible mechanisms of MKP-1 inhibition and selectivity [80, 81]. The most potent member of this library, compound (**1**), based on IC_50_ measurements is shown in Fig. (**6**). Based on unbiased docking simulations using AutoDock [82], we identified the regions in the vicinity of the active sites of MKP-1, MKP-3, VHR, and Cdc25B that can potentially be involved in competitive inhibition [81]. The comparative analysis of the surface properties of these four DSPs at the identified regions showed that the MKP-1 and MKP-3 are characterized by a more hydrophobic character compared to other DSPs. The higher tendency of these two DSPs to bury a hydrophobic region appears to be in accordance with the relatively higher inhibitory activity of compounds against MKP-1 and MKP-3 observed in *in vitro* assays.

We further performed targeted (or biased) docking simulations in the putative binding site of MKP-1. Results from ensemble modeling of interactions were obtained using a combination of comparative modeling (MODELLER [83]), structure refinement (Sybyl 7.2; Tripos, Inc. St. Louis, MO), and docking tools (GOLD [84]). Using the catalytic domain structures of MKPs in the active state listed in Table **1**, we generated 300 models (conformations) of MKP-1. The objective was to take into consideration the possible structural flexibility (and/or inaccuracy) of the modeled target proteins (encircled region in Fig. **7A**). The two enantiomers of compound **1** were docked five times onto each MKP-1 conformation, resulting in 3,000 docking poses. Analysis of the resulting ensemble of poses to retrieve dominant patterns and identify the most favorable poses revealed the role of the solvent-exposed side-chains of His229 and Phe299 in optimizing the interactions with the inhibitor (Fig. **7B**). These residues presumably mediate the binding of pyrrole carboxamide inhibitors in favor of a geometry that occludes the access of substrates to the catalytic site.

**Fig. (6):**
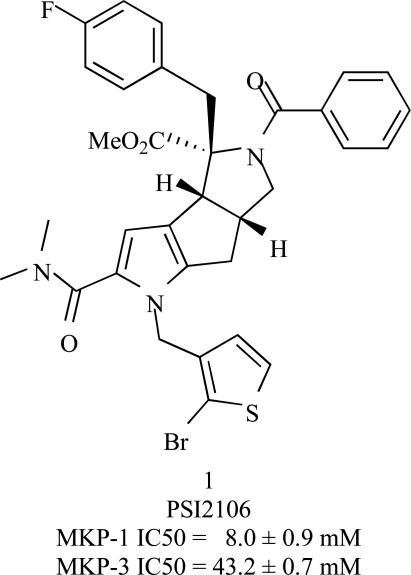
**MKP-1 inhibitor from a focused chemical library [81].** Note the comparable activities (IC_50_ values) of these compounds against MKP-1 and MKP-3.

**Fig. (7):**
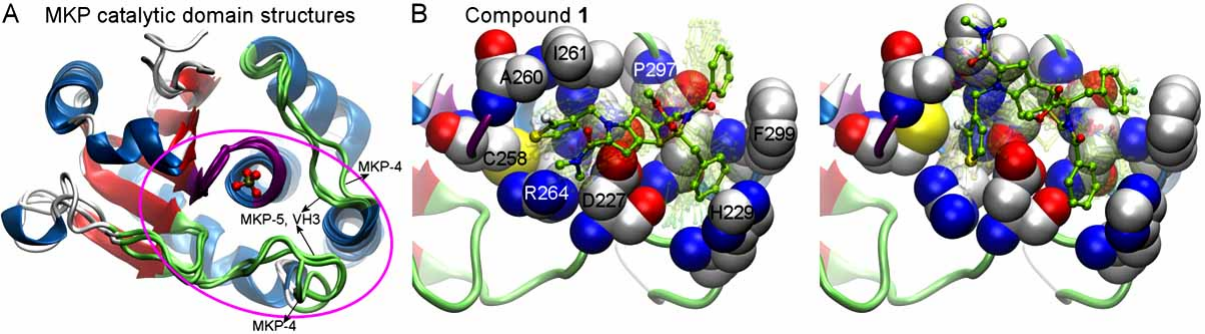
**MKP CD structures and docking solutions for MKP-1 inhibitors. A.** Template structures used in modeling the MKP-1 catalytic domain. The inhibitor docking site is encircled. **B.** Docking solutions for enantiomers of compound (**1**). Note the interactions with H229 and F299, in addition to those with A260, I261 and R264 on the HC*X*_5_R motif. Inhibitors are shown in a ball-and-stick representation with C atoms colored green. Approximately 5% of docking poses in the same bound state/cluster are shown transparently.

These observations suggest some criteria for designing MKP inhibitors. However, achieving selectivity remains a challenge. Sequence comparison between the members of the MKP family shows that MKP-1 shares a high sequence identity with others especially in the active site region (Table **1**). Specifically, Phe299 is highly conserved (see Fig **4**), while the His229 position appears to sustain substitutions to Trp, Asn or Glu, which may impart some selectivity. MKP-1 inhibitors are known to have comparable inhibitory potency against MKP-3 (see the IC_50_ values in Fig. **6**). We note that the MKP-1 His229 residue is substituted by Trp264 in MKP-3. Cross-docking of the above described compounds to an ensemble of MKP-3 models resulted in similar observations, in accordance with their comparable inhibitory activities. These observations highlighted the need for locating other binding sites on MKP-1 for designing selective inhibitors. We also note that these simulations were based on models generated using the active state structures of the MKP catalytic domains listed in Table **1**. Further studies considering the inactive conformation of the active site loop, as well as the accessible conformations predicted by normal mode analysis, are expected to assist in generating more accurate and comprehensive predictions of MKP-inhibitor interactions.

## FUTURE PROSPECTS: INTEGRATED CHEMICAL SYNTHESIS AND VIRTUAL DESIGN

The studies highlighted above, along with others [85, 86], show that noteworthy advances have recently been made in addressing the challenging problem of designing inhibitors for DSPs, although overall progress in this field has been limited by the peculiar structural characteristics of this class of signaling enzymes. An integrated chemical synthesis and structure-based ligand design strategy, along with virtual docking tools that take into account the conformational variability of the target protein, emerge as key components in the advances made to date in this area. While high throughput screening assays provide us with extensive data, these results are often of limited utility unless they are complemented by structure-based, detailed studies of well-defined target proteins and their interactions with small molecules, which in turn, depend on the availability of accurate experimental data. A major bottleneck toward rapid progress in the structure-based discovery of lead compounds, including in particular those for DSPs, is the lack of detailed, complete datasets, combining both structural and binding affinity data with a series of ligands for well-defined target proteins. The collection of such high quality data for a representative set of protein targets of therapeutic significance is likely to provide a benchmarking set that can assist in improving current molecular computational models, methods and software, which in turn, will assist experimental efforts toward drug discovery. It is important to note that the optimal development cycle involves an iterative design-synthesis strategy, whereby new computational hypotheses can be evaluated by specifically prepared synthetic small molecules.

## Figures and Tables

**Fig. (1):**
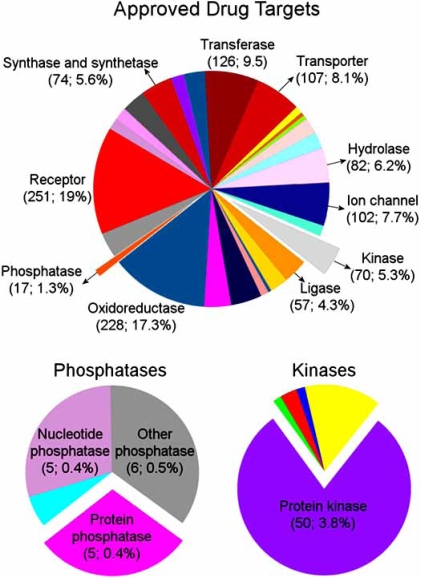
**Distribution of biological targets among approved drugs.** Molecular functions of 1321 approved human drug targets retrieved from DrugBank [13] are assigned using the PANTHER classification system [14]. Phosphatase and kinase slices are enlarged in two separate pie charts. We note that a much smaller number of protein phosphatases are validated as targets compared to protein kinases. The numbers in parentheses represent the numbers of target proteins in each category and their fractional contribution to the entire set of approved drug targets. There are no dual-specificity phosphatases approved as drug targets. MKP-3 is listed in the DrugBank as an experimental drug target.

**Fig. (5):**
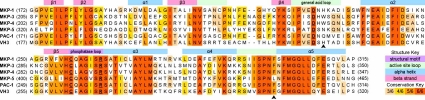
**Sequence alignment of the catalytic domain of MKP-1 against other MKPs with known high-resolution structure.** The small triangles indicate the sequence positions of the two residues (His229 and Phe299 in MKP-1) implicated in inhibitor binding.

**Table 1:** Known DSP Catalytic Domain Structures and their Sequence Identities

	Catalytic domain structures			% pairwise sequence identity among MKPs *					
Name	PDB ID	Res. (Å)	State	MKP-1	MKP-3	MKP-4	MKP-5	PAC-1	VH3
**MKP-1**	–	–	–	–	47.26	46.58	41.78	73.97	64.38
**MKP-3**	1MKP [45]	2.35	Inactive	58.06	–	80.14	47.26	47.95	43.84
**MKP-4**	2HXP	1.83	Active	54.84	96.77	–	46.58	47.95	43.84
**MKP-5**	1ZZW [47]	1.60	Active	54.84	54.84	58.06	–	42.47	34.93
**PAC-1**	1M3G [46]	NMR	Inactive	77.42	51.61	48.39	48.39	–	57.53
**VH3**	2G6Z [48]	2.70	Active	70.97	48.39	45.16	41.94	58.06	–
**Cdc25A**	1C25 [24]	2.30	–	–	–	–	–	–	–
**Cdc25B**	1QB0 [25]	1.91	–	–	–	–	–	–	–

* Upper triangular entries refer to the sequence identity percentages at the catalytic domains; lower triangular entries to those at the active site region of the catalytic domain. The corresponding multiple sequence alignment is given in Fig. (**5)**.
